# Editorial: New insights into vascular language disorders

**DOI:** 10.3389/fnhum.2022.1087848

**Published:** 2023-01-06

**Authors:** Jean-Marie Annoni, Ana Ines Ansaldo, Swathi Kiran, Paola Marangolo

**Affiliations:** ^1^Neurology Unit, Department of Neuroscience and Movement Science, Faculty of Science and Medicine, University of Fribourg, Fribourg, Switzerland; ^2^Laboratoire de Plasticité Cérébrale Communication et Vieillissement-Centre de Recherche de l'Institut Universitaire de Gériatrie de Montréal (CRIUGM) and École d'Orthophonie et d'Audiologie, Faculté de Médecine, Université de Montréal, Montreal, QC, Canada; ^3^Aphasia Research Laboratory, Department of Speech, Language and Hearing Sciences, Boston University College of Health and Rehabilitation Sciences: Sargent College, Boston, MA, United States; ^4^Department of Humanities Studies, University of Naples Federico II, Naples, Italy

**Keywords:** aphasia (language), recovery, neural networks, stroke, plasticity, speech therapy

This special issue is built around the topic of neuroplasticity-induced changes in functional networks as a result of language recovery after brain lesions. As we know, Aphasia knowledge has first relied on the seminal clinical-anatomical works describing the “zone du language” and attached bundles (Dejerine, 1901). In recent decades, the brain-language link has integrated theories of linguistic and cognitive models of language, data from structural and functional biomarkers, and principles of short -and long- term plasticity. These findings have led to a refinement of aphasia comprehension, and of its mechanisms of recovery after brain lesions. This issue proposes studies investigating factors related to language recovery in people with aphasia (PWA). The particularity of this special issue is its strong clinical approach to brain-language relationship, integrating aphasia clinical profiles and resilience, with neurobiological markers.

A first article tackles the effect of language impairment related to focal lesions on global brain activity. Enlarging the concept that a focal brain lesion could significantly modify global functional networks. Dalton et al. showed not only global slowing of spectral resting state EEG in PWA but also quantitative correlations between such slowing and language data. Such data are promising for sensitive electrophysiological correlations of cognitive functioning and change in chronic PWA.

Then, a series of articles approach, through clinical-neuroimaging investigations, the role of specific white matter structures in language impairment. They offer interesting insights on the role of subcortical reorganization in interpreting different aphasia symptoms and their recovery. Ivanova et al. address the specific contributions of the Arcuate Fasciculus (AF) segments to language on a cohort of chronic left hemispheric stroke PWA. They found that the left long segment of the AF contributes to naming abilities, while the anterior part to fluency and naming, and the posterior one to comprehension. These results highlight the important contributions of the AF fiber pathways to language impairments and point to the posterior segment of this tract as being most crucial for supporting residual language abilities. Kourtidou et al. investigate possible mechanisms of right hemisphere compensation by studying chronic PWA. Using diffusion tensor imaging (DTI) and correlating language performance with radial diffusivity (RD) in the right hemisphere homologs of damaged left structure, they argue in favor of compensatory roles of the right hemisphere tracts in language functions when the LH networks are disrupted. Finally, in the study by Lwi et al. on a group of chronic left post-stroke PWA, the authors confirm the critical role of intact posterior left middle temporal gyrus in the recovery of auditory comprehension. The study also highlights that spontaneous recovery of auditory comprehension can continue beyond the first-year post-stroke, independently of age and gender. The role of the cerebellum as part of the language network, and in aphasia recovery is addressed in an innovative multiple case study by Geva et al.. Detailed analysis of four patients with cerebellar lesions suggests that focal damage to the right posterior cerebellum, particularly to lobule IX, may induce sentence comprehension and production impairments, demonstrating the contribution of such structure to language processing.

The last two studies focused on more general principles of aphasia recovery. In a Meta- Analysis using an original method of activation likelihood estimates across 44 published fMRI and PET studies to characterize the functional reorganization in left post-stroke PWA, LaCroix et al. found that PWA engage left perilesional regions during language comprehension and right hemispheric areas in language production, relying generally on similar cognitive-linguistic neural resources for language as controls. Finally, a last behavioral study by Masson-Trottier et al. addresses the role of bilingualism in aphasia recovery and more specifically, the effect of French Phonological Component Analysis—a training approach which uses phonological cues associated with the target word to elicit naming. The authors compared monolingual and bilingual PWA performance both at the linguistic and cognitive control level to examine the structural impact of the left hemispheric lesion location on right hemisphere structural data. Results show that bilinguals improved more than monolinguals in picture naming and narrative discourse tasks potentially due to enhanced cognitive control abilities, which could be supported by right hemisphere neural reserve.

In sum, this special issue points to the variety of linguistic and non-linguistic networks sustaining recovery following brain damage, providing evidence for their potential role in language recovery. Please enjoy the reading.

## Author contributions

J-MA wrote the first draft and reviewed the manuscript. AA and PM introduced their own input and reviewed the manuscript. SK checked and edited the manuscript. All authors contributed to the article and approved the submitted version.

## References

[B1] Dejerine J. (1901). Anatomie Des Centres Nerveux Tome II. ed J. Rueff. Paris: Rueff.

